# Supplementary material for: JAZF1, A Novel p400/TIP60/NuA4 Complex Member, Regulates H2A.Z Acetylation at Regulatory Regions
Source: Int J Mol Sci. 2021 Jan 12;22(2):678. doi: 10.3390/ijms22020678 (PMC7826843; doi:10.3390/ijms22020678)
Supplement: Supplementary file 1 [file ijms-22-00678-s001.zip › ijms-1062149 SI/Supplementary Figures.docx]

Supplementary Figures

**Supplementary Figure 1: JAZF1 depletion affects expression of genes involved in ribosome biogenesis. (A)** JAZF1 expression analysis by RT-qPCR two days after control (siNTC, two and three replicates) or JAZF1 (siJAZF1, two and three replicates) knockdowns in HeLa Kyoto cells using two independent JAZF1 primer pairs (JAZF1_1, light brown; JAZF1_2, dark brown) of batch one (top) and batch two (bottom) experiments. All data were normalized to HPRT1 expression and depicted as fold change expression compared to control knockdowns. Error bars indicate SD of three replicates. Shown is one of more than three biological replicates showing similar results. See Figure 3 and (B) for comprehensive RNA-seq data analyses. **(B)** Principle component analysis (PCA) of RNA-seq data of two batches (knock-downs on different days; experiment 1: circles, experiment 2: squares) of control (siNTC, two and three biological replicates, red) or JAZF1 (siJAZF1, two and three biological replicates, blue) knockdowns. Notice, that while batches show strong variations, biological effects observed upon JAZF1 depletion in comparison to controls are highly similar between batches and replicates, indicating similar biological effects. See also Figure 3 for combined RNA-seq analyses of batches and replicates. **(C and D)** Dot plots after gene set enrichment analysis (GSEA) for the sub-ontology (GO database) cellular compartment (CC; (**B**)) or biological pathways (BP; (**C)**). Shown are the three pathways with the lowest adjusted p-value and all significant (adjusted p-value < 0.1) pathways that are associated with ‘Ribosome / Ribosome Biogenesis’. Dot size represents the number of genes that belong to each pathway; colors indicating the adjusted p-value; position on the x-axis shows the normalized enrichment score. See corresponding GSEA plot of ‘Ribosome’ (KEGG database) in Figure 3C.


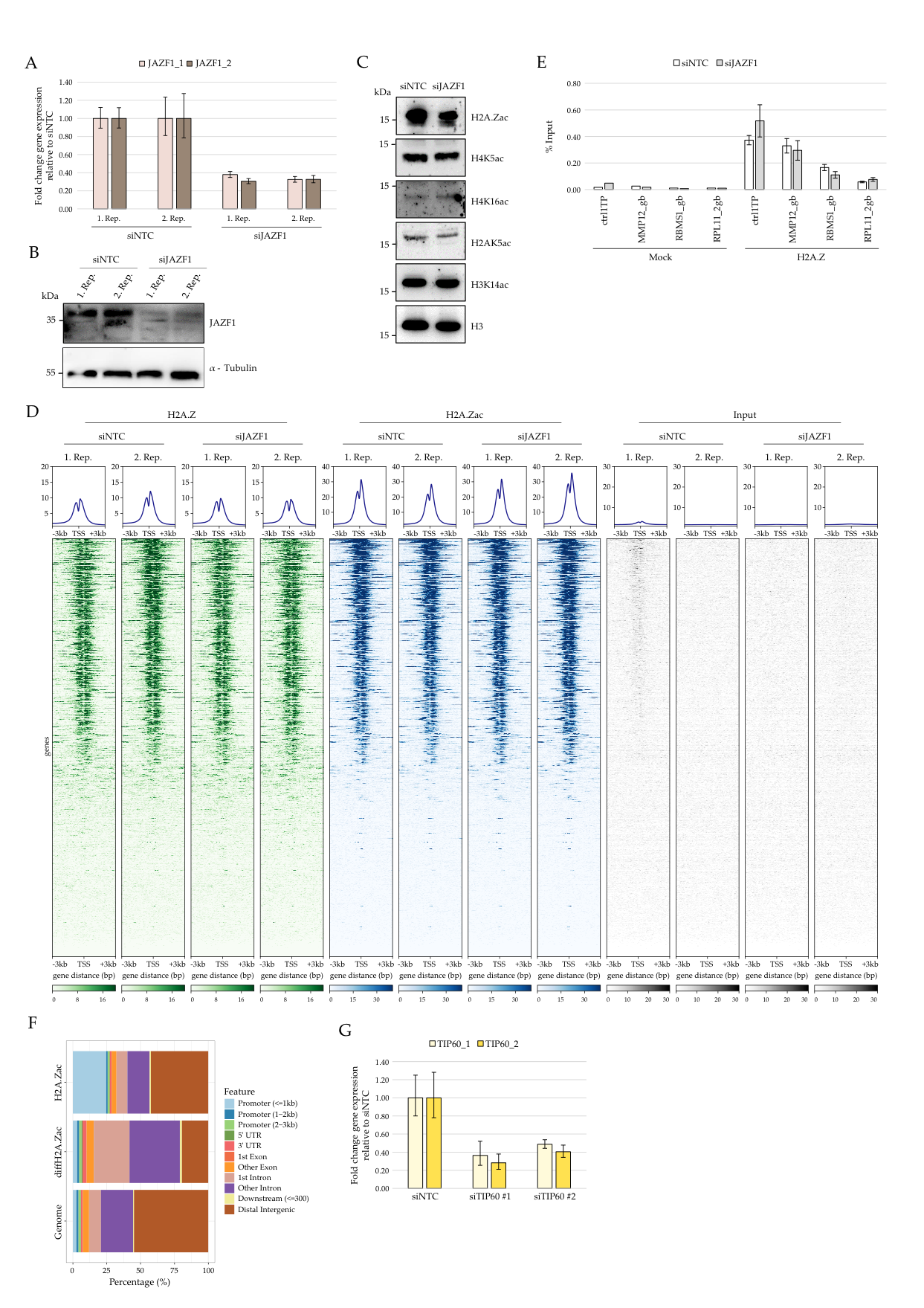


**Supplementary Figure 2: JAZF1 depletion does not change global H2A.Z or H2A.Zac levels. (A)** JAZF1 expression analysis by RT-qPCR two days after control (siNTC, two replicates) or JAZF1 (siJAZF1, two replicates) knockdowns in HeLa Kyoto cells using two independent JAZF1 primer pairs (JAZF1_1, light brown; JAZF1_2, dark brown). All data were normalized to HPRT1 expression and depicted as fold change expression compared to control knockdowns. Error bars indicate SD of three replicates. Shown is one of more than three biological replicates showing similar results. **(B)** Immunoblot analysis of whole cell extracts after JAZF1 knockdown described in (A). Antibody staining against α-Tubulin served as loading control. **(C)** Immunoblots analyses of acid-extracted histones using different histone modification antibodies after JAZF1 knockdown as described in (A). Anti-H3 antibody was used as loading control. Notice no change in histone modification levels comparing histones extracted from control (siNTC) versus JAZF1 (siJAZF1) knockdowns is seen. **(D)** ChIP-seq density heatmap of global H2A.Z (green), H2A.Zac (blue) and Input (gray) upon control (siNTC) or JAZF1 (siJAZF1) knockdowns (two replicates) at transcriptional start sites (TSS) of genes. Color intensities represents normalized and globally scaled tag counts. Notice that at promoter regions H2A.Z and H2A.Zac peaks do not change upon JAZF1 reduction and compare to differential H2A.Zac sites at some enhancer regions as depicted in Figure 4. **(E)** ChIP-qPCR verification of unchanged H2A.Z levels at three diffH2A.Zac sites (see Figure 4F) upon JAZF1 (siJAZF1, gray) compared to control knockdown (siNTC, white) using H2A.Z or no antibody (mock). Shown is the respective enrichment as percentage of input signals. Error bars represent SD of three replicates. Shown is one out of three biological replicates showing similar results. **(F)** Feature enrichment plot of global H2A.Zac and diffH2A.Zac peaks upon JAZF1 knockdown in comparison to complete genome. Notice enrichment of diffH2A.Zac peaks in first and other introns. See also Figure 4e for ChromHMM characterization showing state enrichment of diff H2A.Zac in regulatory regions within gene bodies. **(G)** TIP60 expression analysis by RT-qPCR two days after control (siNTC) or TIP60 (two independent siRNAs: siTIP60 #1 or #2) knockdowns in HeLa Kyoto cells using two independent TIP60 (TIP60_1, light yellow; TIP60_2, dark yellow) primer pairs. All data were normalized to HPRT expression and depicted as fold change expression compared to control knockdowns. Error bars indicate SD of three replicates. Shown is one out of two biological replicates showing similar results. See corresponding ChIP-qPCR analysis in Figure 4F.
