# Supplementary figures and images for: JAZF1, A Novel p400/TIP60/NuA4 Complex Member, Regulates H2A.Z Acetylation at Regulatory Regions
Source: Int J Mol Sci. 2021 Jan 12;22(2):678. doi: 10.3390/ijms22020678 (PMC7826843; doi:10.3390/ijms22020678)

Supplementary Figure 1

A

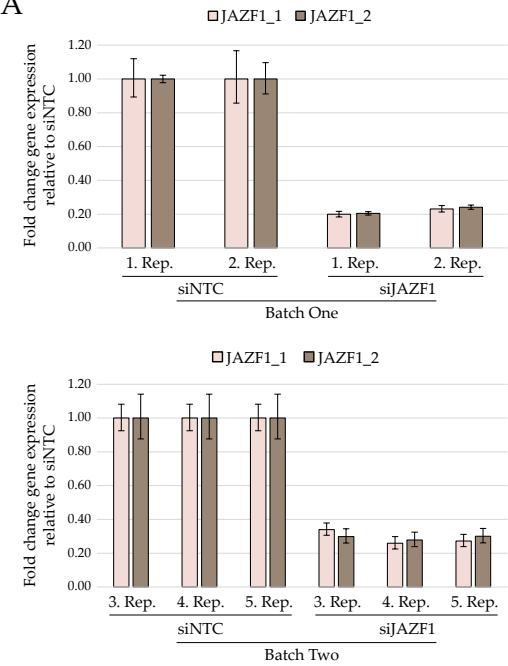

B

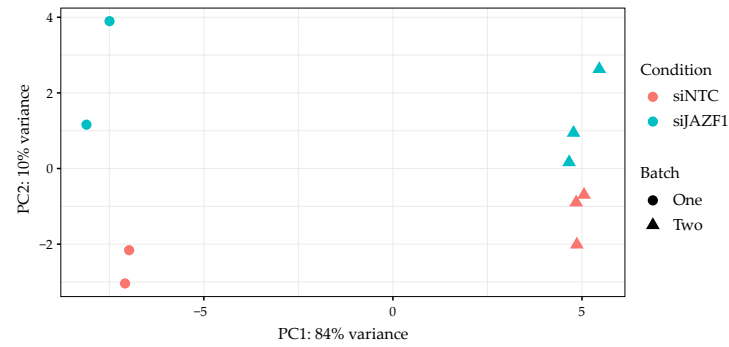

C

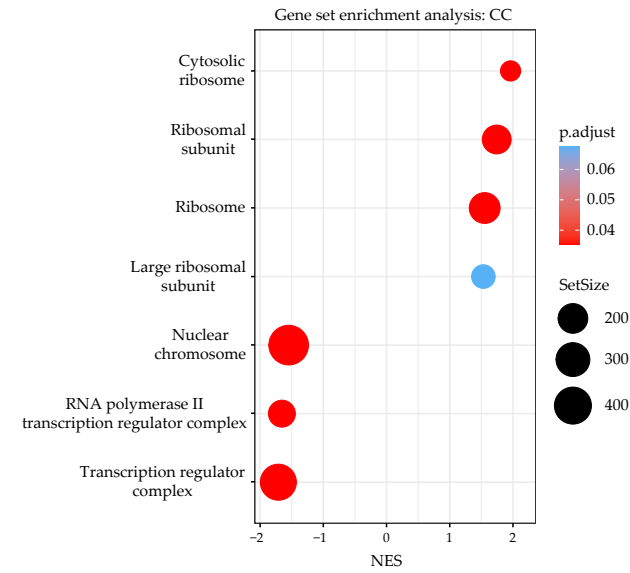

D

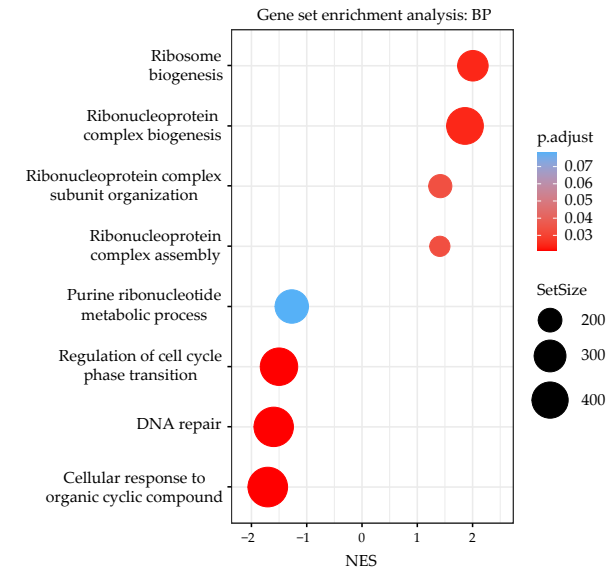

Supplement: Supplementary file 1 [file ijms-22-00678-s001.zip › Figure 1 Supplement.pdf]

Supplementary Figure 2

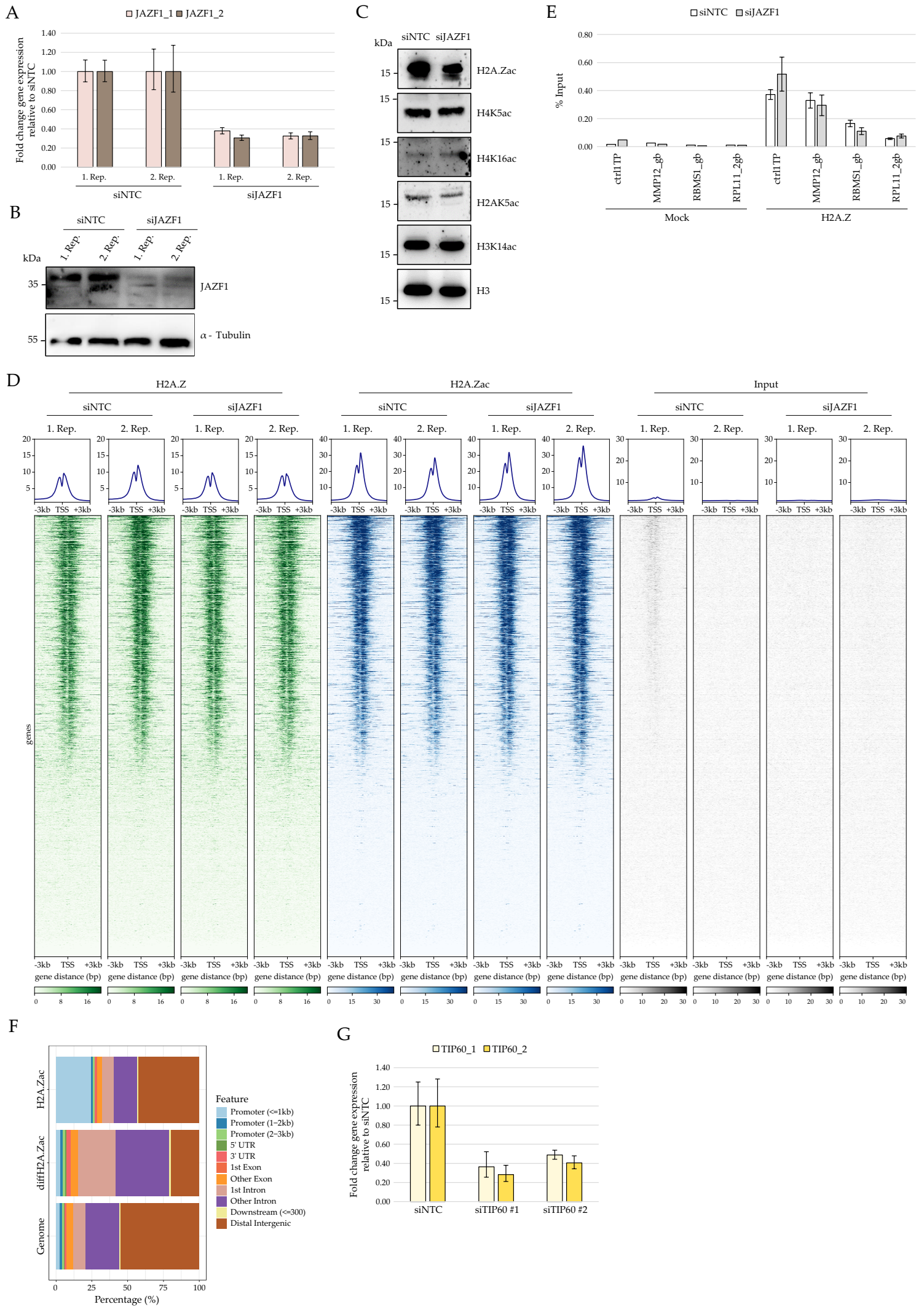

Supplement: Supplementary file 1 [file ijms-22-00678-s001.zip › Figure 2 Supplement.pdf]
